# Cryptococcosis with an endobronchial tumor-like growth in an immunocompetent patient

**DOI:** 10.1590/0037-8682-0497-2023

**Published:** 2024-02-05

**Authors:** Felipe Marques da Costa, Bruno Lima Moreira, Milena Tenório Cerezoli, Christina Shiang, Fábio José Haddad

**Affiliations:** 1 Hospital Beneficência Portuguesa de São Paulo. São Paulo, SP, Brasil.

A 60-year-old woman nonsmoker presented with a 3-month history of progressive dyspnea, cough, sputum production, and weight loss. She worked as a librarian in an old building and reported no prior medical history of note.

**FIGURE 1:**
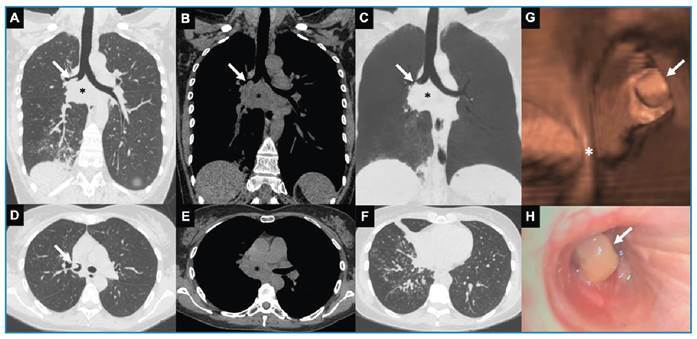
Chest computed tomography (CT) (**A-G**) and bronchoscopy (**H**) performed during the investigation of symptoms. (**A-C**) Coronal oblique images (**A**: lung window; **B**: mediastinal window; **C**: lung window with minimum intensity projection) and (**D-F**) axial images (**D**: lung window; **E**: mediastinal window; **F**: lung window) display a heterogeneous mass/lymph node conglomerate (black asterisk) with hypodense components suggestive of necrosis in the mediastinum at the subcarinal level, with an endobronchial component (white arrow) extending into the right main and intermediate bronchi related to the middle and right lower lobes. Parenchymal abnormalities including consolidation, centrilobular nodules with a tree-in-bud pattern, and subsegmental atelectasis are present in the middle and right lower lobes. CT virtual bronchoscopy (**G**) and fiberoptic bronchoscopy (**H**) reveal an endobronchial lesion with polypoid appearance (white arrow) in the right main/intermediate bronchus (white asterisk: tracheal carina).

**FIGURE 2:**
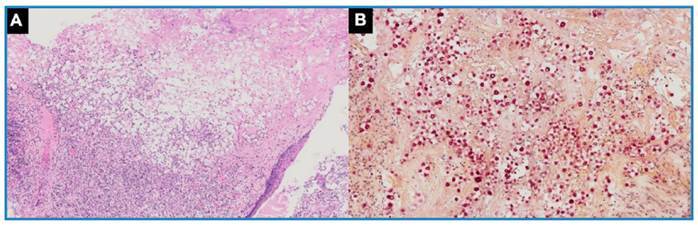
Microscopy of the bronchial biopsy. (**A**) Mucosa with extensive ulceration and necrosis, squamous metaplasia of the epithelium, and expansion of the chorion owing to intense inflammation containing many yeast capsules (hematoxylin and eosin, 2×). (**B**) Mucicarmine stain highlighting fungal capsules rich in polysaccharides, consistent with *Cryptococcus* spp. (5×).

**FIGURE 3:**
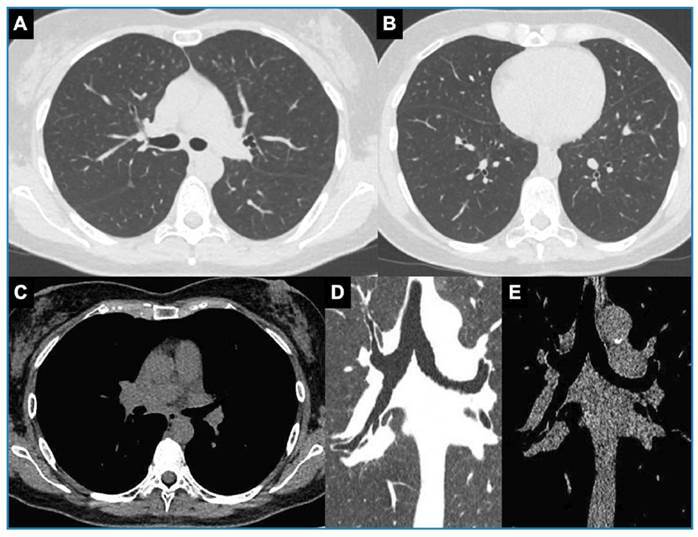
Chest computed tomography after completing fluconazole treatment. Axial images (**A, B**: lung window at two different levels; **C**: mediastinal window) and coronal images (**D**: lung window; **E**: mediastinal window) showing regression of the mass/lymph node conglomerate in the visceral compartment of the mediastinum at the subcarinal station, with a resolution of the endobronchial component and clearance of the parenchymal abnormalities in the right lung.

Physical examination revealed a right-sided expiratory wheeze and an oxygen saturation of 92%. Chest computed tomography (CT) revealed a heterogeneous mediastinal mass/lymph node conglomerate with an associated right-sided endobronchial component, as well as homolateral lung opacities, suggesting a post-obstructive pulmonary infection and/or airway dissemination of an infectious agent (Figure 1A-G). The patient was treated with intravenous ceftriaxone. Additional tests included bronchoscopy (Figure 1H) with bronchoalveolar lavage (BAL) and endobronchial biopsy; and serology. The histopathology of the bronchial biopsy revealed findings consistent with cryptococcosis (Figure 2), and serum antigen testing was positive (1/256) for *Cryptococcus neoformans*. No lumbar puncture was performed because the patient tested HIV negative and had no neurological symptoms. She was treated with fluconazole (400 mg/day for 10 months), which resulted in clinical and radiological improvement (Figure 3).

Bronchopulmonary cryptococcosis (usually caused by *Cryptococcus neoformans* or *Cryptococcus gattii*) is commonly acquired through inhalation of spores from soil contaminated with bird droppings^1^ ^,^ ^2^. In immunocompetent hosts, imaging often reveals patchy consolidation or a solitary pulmonary nodule or mass. Endobronchial tumor-like growth is rare^1^ ^-^ ^5^. Diagnosis is established by identifying encapsulated yeast in sputum, BAL, and tissue samples, and elevated serum antigen titers^4^. Treatment includes endoscopic or surgical removal of mass lesions, and administration of antifungal agents such as amphotericin B, flucytosine, fluconazole, itraconazole, or voriconazole^3^ ^,^ ^5^.
